# Nanoparticle Size Effect on Water Vapour Adsorption by Hydroxyapatite

**DOI:** 10.3390/nano9071005

**Published:** 2019-07-12

**Authors:** Urszula Szałaj, Anna Świderska-Środa, Agnieszka Chodara, Stanisław Gierlotka, Witold Łojkowski

**Affiliations:** 1 Institute of High Pressure Physics, Polish Academy of Sciences, Sokołowska 29/37, 01-142 Warsaw, Poland; 2 Faculty of Materials Engineering, Warsaw University of Technology, Wołoska 41, 02-507 Warsaw, Poland

**Keywords:** adsorption, hydroxyapatite, nanoparticles, size effect, size dependent, water layers, humidity, ambient conditions

## Abstract

Handling and properties of nanoparticles strongly depend on processes that take place on their surface. Specific surface area and adsorption capacity strongly increase as the nanoparticle size decreases. A crucial factor is adsorption of water from ambient atmosphere. Considering the ever-growing number of hydroxyapatite nanoparticles applications, we decided to investigate how the size of nanoparticles and the changes in relative air humidity affect adsorption of water on their surface. Hydroxyapatite nanoparticles of two sizes: 10 and 40 nm, were tested. It was found that the nanoparticle size has a strong effect on the kinetics and efficiency of water adsorption. For the same value of water activity, the quantity of water adsorbed on the surface of 10 nm nano-hydroxyapatite was five times greater than that adsorbed on the 40 nm. Based on the adsorption isotherm fitting method, it was found that a multilayer physical adsorption mechanism was active. The number of adsorbed water layers at constant humidity strongly depends on particles size and reaches even 23 layers for the 10 nm particles. The amount of water adsorbed on these particles was surprisingly high, comparable to the amount of water absorbed by the commonly used moisture-sorbent silica gel.

## 1. Introduction

Exploitation of the specific properties of nanomaterials, which are not observed in their microcrystalline counterparts, is a fundamental basis of nanotechnology [1]. Due to these properties, nanoparticles are increasingly applied as additives, modifiers of composite materials, reaction catalysts [2,3], nano-solders [4,5], sensors [6,7,8], coatings [9,10] and materials for medical applications [11,12,13,14,15,16]. Over the past few decades, millions of tons of nanoparticles have been produced [17]. Nanoparticles dispersed in a base material may act as the nucleus of new phases, facilitate its hydration, and reinforce and compact the microstructure, thus reducing the material’s porosity [18]. The large specific surface area of nanomaterials leads to sorption processes on their surfaces. As a result, these materials can bind water contained in the air even during production processes. Depending on the application of a given nanomaterial, the water adsorbed on the nanoparticle surface may be either beneficial or unfavourable [19].

Nanotechnology and nanomaterials are very promising for modern medicine. A frequently applied bio-nanomaterial is hydroxyapatite (HAP), which is a mineral that naturally occurs in the form of calcium apatite. Composites composed of HAP nanocrystals and biocompatible polymers are widely used in orthopaedics [20,21,22] and dentistry [23,24]. Dispersing the hydroxyapatite in a polymer reinforces its mechanical properties and improves osteoinductiveness and biocompatibility [25,26,27]. Water adsorption by hydroxyapatite attracted considerable interest since the 1960s/1970s due to the application of this material in dentistry, and sorption processes occurring on microcrystalline, synthetic, and natural hydroxyapatite were described [28,29,30,31]. The ionic nature of HAP was indicated as the cause of strong bonds forming between polar adsorbates and the hydroxyapatite surface [28]. To verify that theory, Dry and Beebe [28] researched the adsorption heat of water and ethanol on microcrystalline hydroxyapatite and determined the adsorption isotherms. It was found that both types of adsorbates were bonded on the HAP surface by hydrogen bonds. In addition, the water molecules were bonded by means of a single hydrogen bond. After covering the surface of microcrystalline hydroxyapatite with one layer of water, it was possible to add another layer of molecules. Dry and Beebe emphasised that, in the case of microcrystalline hydroxyapatite, it was very difficult to distinguish physical adsorption of water molecules from chemical adsorption.

Rootare and Craig [29] examined sorption processes for microcrystalline HAP with different specific surface areas of 3, 22, and 70 m^2^/g. Water adsorption isotherms and adsorption heat at a temperature of 300 °C were calculated, and the number of water molecule layers was established to be 8, 9, and 15, respectively. According to Rootare and Craig, the first layer of water molecules was bonded by HAP in a chemisorption process, whereas successive layers were added in a physical adsorption process. Holmes and Beebe [30] found that the quantity of water bonded to the microcrystalline HAP surface depended on the sample history.

Wade et al. [31] attempted to determine the impact of relative air humidity on hydroxyapatite compactness. The moisture content of granulated hydroxyapatite was determined using thermogravimetric analysis (TGA) within the temperature range from 25 °C to 450 °C. The total mass loss of the sample stored at a temperature of 20 °C and with RH = 30% amounted to 5.26% ± 0.04%. According to the authors, about 0.7% of the weight was loosely bounded water, which was lost at temperatures from 20 °C to 60 °C. About 0.5% of the mass was lost by degassing water from the surface of the granulated HAP at temperatures from 60 °C to 100 °C. At temperatures from 100 °C to 450 °C, approximately 4% of the sample weight was lost as a result of the desorption of water adsorbed by the chemisorption process.

Hydroxyapatite with a nanometric particle size is attracting considerable interest. Hydroxyapatite nanocrystals contained in human bones are 5–20 nm wide and 60 nm long [32]. Therefore, synthetic nano-hydroxyapatite is particularly valuable for use in regenerative medicine since its particle size is similar to that of natural HAP. Nano-HAP is also widely used in toothpaste, cosmetics, and products that fill wrinkles (in conjunction with hyaluronic acid) [33,34].

In the fields of pharmacy and regenerative medicine, control of the hydrophilic properties of materials is very desirable [32,35,36,37]. For example, the addition of a hydrophilic filler to hydrophobic Polylactic acid PLA allows nano-fibres to be produced from this hydrophilic composite. The addition of a hydrophilic filler, e.g., hydroxyapatite, also improves the mechanical properties and biocompatibility of the implant [38,39,40,41]. On the other hand, too much additive can cause significantly decreased tensile strength of PLA/HA composites and faster composite degradation [42,43]. Another important example is the change in the tensile strength of ibuprofen and paracetamol tablets with moisture content. A low moisture content (in the range 1% to 4% w/w) in tablets increases the tensile strength [40,44,45,46]. Exceeding the limit value of the water content in the tablet results in a significant reduction in tensile strength and disintegration [47]. An understanding of sorption properties is needed to enable control over the quantity of water that is adsorbed by a given nanomaterial.

We studied the sorption properties of GoHAP™ with particle sizes of 10 and 40 nm [48] in order to compare these adsorption properties of nanomaterials and their microcrystalline counterparts. GoHAP™ nanoparticles were produced by the hydrothermal method of microwave synthesis in the MSS-2 reactor, which is a unique method that allows nanoparticles with a controlled particle size to be obtained. This technique can be applied not only to hydroxyapatites, but also to metal oxides such as ZnO and ZrO_2_ as well as doped metal oxides. Microwave synthesis enabling control of particle size has been precisely described [32,49,50,51,52,53,54,55]. Due to the high surface area of such small nanoparticles, on the order of 200 m^2^/g, high water absorption can be expected, which creates a considerable technological challenge [56,57]. Further, a high radius of curvature of the nanoparticle surfaces and surface steps may induce differences in interaction energies compared with those for relatively flat microcrystalline particles [35]. To the best of our knowledge, no research into the processes of water adsorption from air by nano-hydroxyapatite as a function of particle size has been presented in the literature to date. Therefore, our results are also useful for comparing the adsorption properties of nanomaterials and their microcrystalline counterparts. 

## 2. Materials and Methods

All the tests presented in this paper were performed at an accredited testing laboratory, No. AB 1503, according to the ISO 17025:2005 standard.

### 2.1. Substrates

The reagents were used in the “as received” condition: calcium hydroxide (Ca(OH)_2_, pure); orthophosphoric acid (H_3_PO_4_, 85 wt % solution, analytically pure); sodium chloride (NaCl, pure). All reagents were purchased from Chempur (Piekary Sląskie, Poland). Deionized water (specific conductance <0.1S/cm, HLP 20UV, Hydrolab, Straszyn, Poland) was used for the synthesis and investigation of kinetics of the water vapour adsorption.

### 2.2. Synthesis of Hydroxyapatite Nanoparticles

Nano-hydroxyapatite with the trademark GoHAP^TM^ was used. The nanoparticles of GoHAP™ hydroxyapatite used in the tests were obtained according to the procedures of the Laboratory of Nanostructures, Polish Academy of Sciences [32,48]. This method of hydroxyapatite synthesis enables control of the size of GoHAP^TM^ nanoparticles within the range 8 ± 2 to 43 ± 4 nm. In the present work, GoHAP™ with particle sizes of 10 and 40 nm were examined and are referred to as GoHAP-1 and GoHAP-6, respectively. Two methods were applied for the synthesis of GoHAP™. The hydroxyapatite sample with the particle size of 10 nm, named GoHAP-1, was obtained with the use of the precipitation method: namely, 10.3458 g of Ca(OH)_2_ was poured into 450 mL of deionised water. Subsequently, the volume of 5.7 mL of H_3_PO_4_ was added to the water suspension of Ca(OH)_2_ by dosing the acid using a digital burette (SI Analytics, Titronic universal, TZ3260, Mainz, Germany). While adding Ca(OH)_2_ to deionised water and dosing the acid, the suspension was intensively stirred using the magnetic stirrer (SLR, SI Analytics, Mainz, Germany) at room temperature (RT). The product, i.e., water suspension of GoHAP™ nanoparticles, was obtained after 30 min of stirring. The reaction of obtaining GoHAP™ is presented in the following Equation (1).

10 Ca(OH)_2_ + 6 H_3_PO_4_*RT* → Ca_10_(PO_4_)_6_(OH)_2_ (↓) + 18 H_2_O (1)

The hydroxyapatite sample with the particle size of 40 nm, referred to as GoHAP-6 in this publication, was obtained as a result of hydrothermal heating of water suspension of GoHAP-1 in the MSS2 microwave reactor (3 kW, 2.45 GHz, IHPP PAS (Warsaw), ITeE-PIB (Radom), ERTEC (Wroclaw), Poland) [58,59]. The following parameters of the microwave hydrothermal heating were applied: power = 3 kW, duration = 1,200 seconds, pressure = 20 bar (equilibrium temperature ∼220 °C). The suspensions of GoHAP™ nanoparticles after decantation were frozen rapidly using liquid nitrogen and dried in the freeze dryer (Lyovac GT-2, SRK Systemtechnik GmbH, Riedstadt, Germany).

### 2.3. Measurement of Density and Specific Surface Area

The specific surface area (SSA) of GoHAP™ was determined by the BET (Brunauer–Emmett–Teller) adsorption method, in accordance with the ISO 9277:2010 standard. The Gemini 2360 surface analyser by Micromeritics was used in the tests. The skeleton density of the samples was measured using the AccuPyc II 1340 helium pycnometer by Micromeritics, in accordance with the ISO 12154:2014 standard. Before the SSA and density measurements, the GoHAP™ was desorbed in a desorption station (Micromeritics, FlowPrep 06) at a temperature of 150 °C for 2.5 h in a helium stream. Based on the experimentally determined SSA and skeleton density, the mean diameter of GoHAP™, also known as the Sauter mean diameter (SMD), was calculated using Equation (2), with the assumption of a spherical shape. The SMD of GoHAP-1 (10 nm) and GoHAP-6 (40 nm) are 10 and 40 nm, respectively. Table 1 presents the SSA, skeleton density, and Sauter mean diameter of the samples.

(2) $$Sauter\;mean\;diameter\;\left(SMD\right)=\frac{A}{SSA\cdot 10^{18}\cdot \rho \cdot 10^{-21}}\;\left(nm\right)$$

where *SMD* is the Sauter mean diameter of nanoparticle (nm); *A* is a shape factor, equal to 6 for the sphere; *SSA* is the specific surface area (m^2^/g); and *ρ* is the density (g/cm^3^). The average pore width was calculated by the Barrett–Joyner–Halenda (BJH) method using adsorption isotherms at the P/P_0_ range of 0.001–0.99. The Adsorption Isotherm was determined using the helium pycnometer at the temperature of 24 ± 1 °C (ISO 12154:2014, AccuPyc II 1340 FoamPyc V1.06, Micromeritics, Atlanta, GA, USA). The total pore volume of samples was estimated from the amount of adsorbed nitrogen at P/P_0_ = 0.9896. The obtained data of the adsorption isotherms were analysed by us using the MicroActive software V4.03 (Interactive Data Analysis Software, Micromeritics). The results of pore width measurements, total pore volume measurements and adsorption isotherm can be found in the Supplementary Materials file (Figure S1, Table S1).

### 2.4. X-Ray Powder Diffraction

The phase purity and the crystallite size were tested by means of X-ray diffraction (XRD) using an X-ray powder diffractometer by Panalytical, model X’Pert PRO equipped with a copper anode (Cu Kα1) and an ultra-fast PIXcel^1D^ detector. The analysis was performed at room temperature in the range 2θ = 10–100° with a step of 0.03°. The analysis of XRD line profiles was carried out using an analytical formula for polydispersed powders [60,61]. The Nanopowder XRD Processor Demo online tool was used. The diffraction files were placed on the website (http://science24.com/xrd/), where they were processed to extract the size distribution of crystallites. This tool solves sets of equations in several auxiliary spaces at the same time, which allows to analyse XRD data with mutually entangled peaks. Thanks to this method, information on four parameters was obtained: mean crystallite size, error of the mean size of crystallites, dispersion of sizes and error of dispersion of sizes. Therefore, the full curve of the crystallite size distribution with its errors has been presented. For reference the mean crystallite sizes of GoHAP™ were also calculated from the diffraction line widths using the conventional Scherrer method [53]. The size along the “c” crystallographic axis was calculated using the (002) diffraction line and the size along the “a” axis was calculated using the (300) diffraction line.

### 2.5. Morphology Characteristics

The morphology of GoHAP™ was tested using SEM (Ultra Plus, Carl Zeiss Meditec AG, Jena, Germany) with Secondary electrons (SE) detector and TEM (FEI Talos F200X, Thermo Fisher Scientific, Waltham, MA, USA), with the beam energy of 2 keV. An amorphous carbon layer produced using the sputter coater (SCD 005/CEA 035, BAL-TEC, Balzers, Lichtenstein) was deposited on the samples for SEM observations. SEM images were obtained at magnifications of 25,000× and 250,000× and with electron high tension of circa 2 kV. TEM observations were carried out with diffraction contrast using two techniques: bright and dark field. In the dark field technique, nanoparticles were observed using a selected beam that was diffracted at an angle corresponding to the diffraction of electrons on the following families of planes: family {100} of hcp structure of Ca_10_(PO_4_)_6_(OH)_2_ with the fixed lattice a = 9.424(4) Å, c = 6.879(4) Å (space group: P63/m, 176). TEM images obtained using the dark field technique allowed us to determine a histogram of GoHAP-6 nanocrystallite size. This procedure consisted of circumscribing circles about visible crystallites, and the diameters of these circles corresponded to the highest longitudinal dimension of these crystallites. Subsequently, using the scale present in each recorded image, the crystallite size was converted to the actual value expressed in nanometres. ORIGIN PRO ver. 7.5 software was used for obtaining a histogram showing the number of crystallites with a given size. A log-normal curve was fitted to the chart and, based on the position of its maximum, the size of the most frequently occurring crystallites was given.

### 2.6. Thermogravimetric Analysis

A thermogravimetric analysis (TGA) of GoHAP-1 (10 nm) and GoHAP-6 (40 nm) was conducted to study desorption as a function of temperature. The measurement was divided into two stages: (1) recording the change in the mass of the sample subjected to helium flushing for 60 min at room temperature, and (2) recording the change in the mass of the sample heated at a rate of 5 °C/minute to a temperature of 800 °C, also in the helium stream. The tests were performed using the Thermogravimetric Analyzer (TGA) (STA 449 F1 Jupiter, Netzsch, Selb, Deutschland). At the same time, the gases released from GoHAP™ during the thermal treatment were analysed using the QMS 403 C Aëolo quadrupole mass spectrometer by Netzsch. The sorption properties of GoHAP-1 (10 nm) and of silica gel (3–5 mm), a typical material used for air drying, were compared. The silica gel was exposed to an ambient relative humidity of 38 ± 2% and 58 ± 2%. The sample mass increment was recorded in situ, and the kinetic curve and the percentage mass increment after 120 min were determined. The results were compared with the corresponding data obtained for GoHAP-1 (10 nm).

### 2.7. Measurement of Relative Humidity 

The temperature and humidity measurements were performed with the use of the Temperature and Relative Humidity Data Logger (TR300, AMPROBE, Seattle, WA, USA) with the measurement range of relative humidity (RH) from 0 to 100%. The accuracy of this device is ± 3% RH and ± 0.6 °C, while the resolution is 0.1% of RH and 0.1 °C.

### 2.8. Kinetics of the Water Vapour Adsorption

Before measurement, GoHAP™ was desorbed at 150 °C for 2.5 h in the helium flow. Degassed samples with a repetitive mass of approximately 0.5 g, poured loosely into vessels with the same overall dimensions, were placed on the pan of the WAA 100/C/1 (RADWAG) analytical scales. The change in the mass of GoHAP™ exposed to an ambient relative humidity of 26 ± 2% and 38 ± 2% (water activity: 0.26 and 0.38, respectively) was recorded in situ; for GoHAP-1 (10 nm), an additional measurement was performed in the ambient relative humidity of 58 ± 2% (water activity: 0.58). The temperature of all experiments was constant at 24 ± 0.5 °C. In order to obtain a relative humidity of 26% or 38%, the stable relative air humidity prevailing in a given period in the test room was used. The highest degree of relative humidity was achieved by placing a vessel containing a saturated aqueous solution of NaCl in a sealed desiccator cabinet with volume 109 L. The overall dimensions of the desiccator were sufficiently large for holding the scales containing the tested sample. Each time, the tests were performed until the sample was water saturated under the given conditions. This corresponded to stabilisation of the sample mass (adsorption equilibrium). The thickness of the water layer corresponding to the saturated condition was calculated. The kinetic curves of water adsorption were prepared, i.e., the curves of the sample mass change as a function of time of exposure for constant relative humidity and temperature. 

During the analysis of the results, we took into account that the specific surface area of GoHAP-1 (10 nm) was over four times greater than that of GoHAP-6 (40 nm). Therefore, two variants for expressing the mass increment were used to present the kinetic curves of water adsorption: (1) by percentage with reference to the initial sample mass (%), and (2) per 1 m^2^ of the surface of the exposed GoHAP™ (mg/m^2^). The percentage mass increment corresponding to the state of equilibrium is described as adsorption. The mass increment per one metre of the adsorbent surface achieved in the saturated condition is referred to as “specific adsorption” in this paper. Furthermore, an isotherm of water vapour adsorption on the surface of GoHAP-1 (10 nm) and GoHAP-6 (40 nm) was determined. Similarly, as with the kinetics tests, samples with a mass of about 0.5 g were desorbed and subsequently exposed to the ambient conditions characterised by varied water activities: 0.03, 0.24, 0.38, 0.51, 0.62, and 0.97. The desired relative humidity was achieved by moisturising the air adjacent to the scales (0.24 and 0.38) or using sealed desiccators with appropriate substances placed inside them: silica gel (water activity 0.03), a saturated aqueous solution of NaCl (0.51 and 0.62), or deionised water (0.94). The samples were exposed to ambient conditions for 24 h. This period was sufficient to achieve a state of equilibrium in each of the experiment variants. In the cases of water activity 0.24–0.62, the change in the sample mass was recorded in situ. At the extreme conditions of the experiment (water activity 0.03 and 0.94), the samples were weighed cyclically using an external scale. The quantity of water adsorbed by GoHAP™, corresponding to the saturated state under the given conditions, was determined. Based on the obtained results, isotherms of adsorption for GoHAP-1 (10 nm) and GoHAP-6 (40 nm) were determined, i.e., curves of change in the quantity of water adsorbed until equilibrium is reached as a function of the ambient water activity. The degree to which the experimental curve fits the BET isotherm models was checked. Based on the isotherm feature, the number of water molecule layers for the equilibrium state at a given humidity was calculated.

## 3. Results

### 3.1. Material Characterisation

The SSA of GoHAP™ of 10 nm (206 ± 1 m^2^/g) is over four times greater than that of GoHAP™ of 40 nm (49 ± 1 m^2^/g). Additionally, its skeleton density is nearly 10% lower: 2.87 ± 0.01 versus 3.09 ± 0.01 g/cm^3^, respectively (Table 1). We suggest that the reduced GoHAP™ density is the result of a greater fraction of the disordered surface layer of nano-hydroxyapatites in the material volume. We described the effect of the nanoparticle density decrease as a function of its SSA increase previously for the example of ZrO_2_ nanoparticles [62]. 

**Figure 1:**
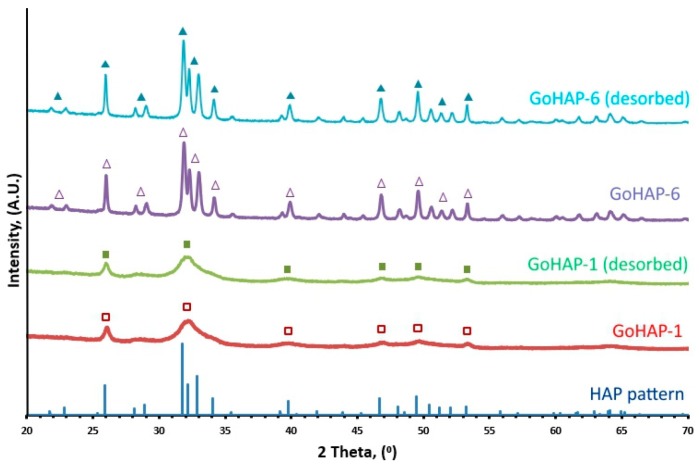
X–ray diffraction line profiles of GoHAP™ with particle sizes of 10 nm and 40 nm before and after desorption.

Figure 1 shows X-ray diffraction line profiles for both sizes of GoHAP™. No phases other than hydroxyapatite were detected. Diffraction profiles for GoHAP™ before and after desorption are essentially identical, which indicates that the desorption process did not impact its crystallinity. The sharper peaks of GoHAP-6 (40 nm) demonstrate that the crystallite size of the latter is larger than that of GoHAP-1 (10 nm). Adsorption and desorption cycles also did not affect the Specific Surface Area and Skeletal Density of GoHAP™ nanoparticles (Table S2).

**Figure 2:**
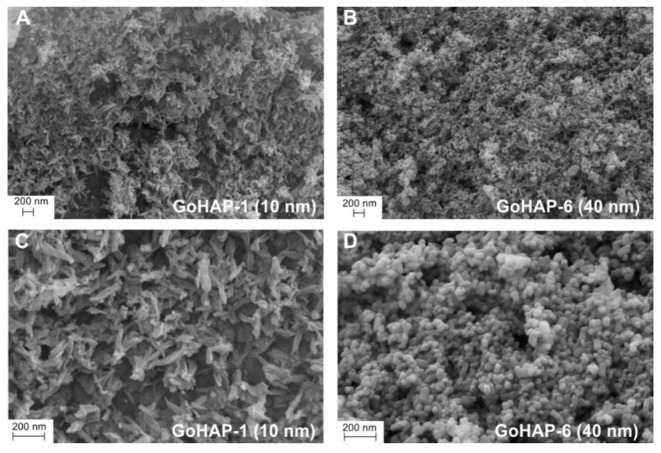
SEM image of GoHAP™ with different particle sizes of 10 nm (**A**,**C**) and 40 nm (**B**,**D**).

Figure 2 presents selected SEM images of GoHAP™. Clusters of spherical objects are visible that have an estimated single constituent size of 10–15 nm for the GoHAP-1 (10 nm) powder and approximately 40 nm for the GoHAP-6 (40 nm) powder.

**Figure 3:**
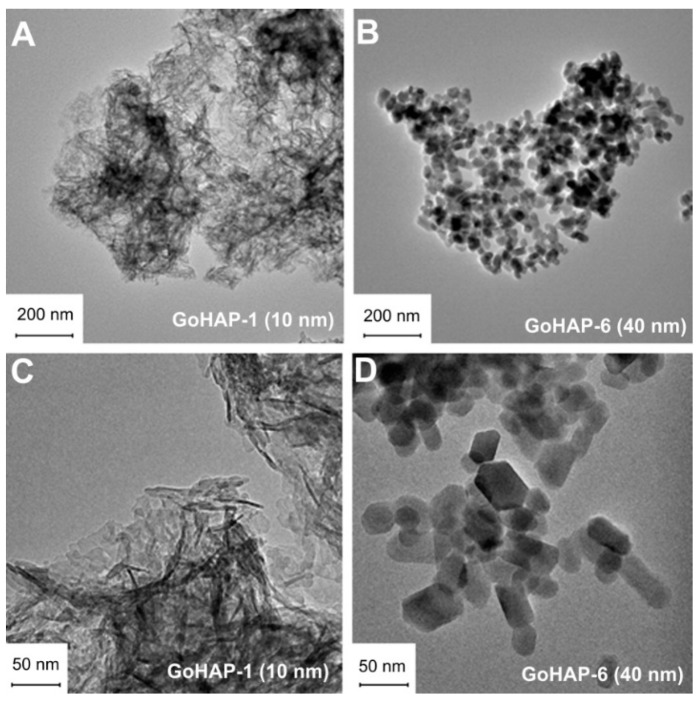
HR TEM image of GoHAP™ with different particle sizes of 10 nm (**A**,**C**) and 40 nm (**B**,**D**).

**Figure 4:**
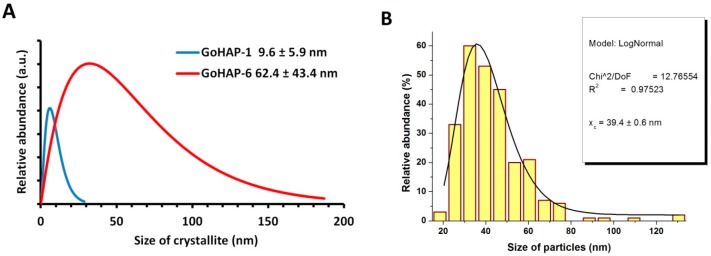
Crystallite size distribution (**A**) based on analysis of the fine structure of the X-ray diffraction line profiles, (**B**) obtained from HR TEM observations.

HR TEM images (Figure 3) obtained by the bright field technique revealed differences in the morphology of GoHAP™. GoHAP-6 (40 nm) has a polyhedron shape, which reflects the hexagonal crystalline structure of hydroxyapatite. TEM images made by the dark field technique enabled the determination of the GoHAP-6 (40 nm) nanoparticle size, and the mean particle size (MPS) based on a fit to the histogram of the log-normal curve is 39 nm (Figure 4B). This value coincides with the 40 nm calculated using the SSA and density parameters (SMD_BET_, Table 1). Based on the GoHAP™ diffraction line profile analysis [60,61], the mean size of the GoHAP-6 (40 nm) crystallites (MCS_XRD_) is 62 ± 43 nm. According to the Scherrer formula, crystallites of GoHAP-6 (40 nm) have a length of 54 nm and a width of 34 nm. In the case of GoHAP-1 (10 nm). Due to the strong tendency to agglomerate, it was not possible to determine the GoHAP-1 (10 nm) mean particle size using microscopic imaging. GoHAP-1 (10 nm) crystallites have a length of 19 nm and a width of 5 nm. An analysis of the GoHAP™ diffraction line profile [60,61] revealed that the mean size of the crystallite (MCS_XRD_) GoHAP-1 (10 nm) was 10 ± 6 nm. Table 1 presents a summary of the quantitative nanoparticle characterisation. The obtained results confirmed the previously reported particle size and shape differences between the two types of GoHAP™ [32].

### 3.2. Examination of Water Adsorption Process

**Figure 5:**
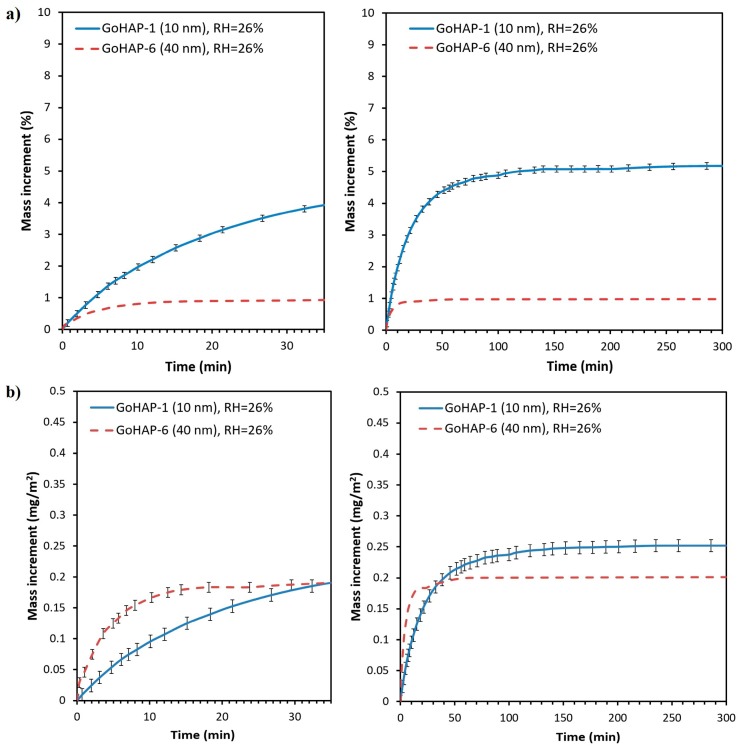
Kinetic curves of water adsorption on GoHAP-1 (10 nm) and GoHAP-6 (40 nm) with an ambient relative humidity of 26%: (**a**) percentage mass change, (**b**) mass increment per 1 m^2^ of GoHAP™.

Regardless of the experimental conditions, the kinetic curves of water adsorption from air by GoHAP™ have a similar shape and are composed of three sequential stages: (1) a rapid, nearly linear mass growth; (2) a gradually inhibited process; and (3) the state of equilibrium and stabilised sample mass. The saturated condition is reached no longer than 200 min after the beginning measurement. Over 50% of the equilibrium water mass is adsorbed during the initial 10 min of exposure. The results of adsorption kinetics tests are summarised in Table 2, and the curves obtained for GoHAP-1 (10 nm) and GoHAP-6 (40 nm) for the ambient relative humidity of 26 ± 2% are presented in Figure 5. The set of curves recorded for GoHAP-1 (10 nm) at three different experimental conditions (water activity: 0.26, 0.38, and 0.58) are presented in Figure 5.

With an ambient water activity of 0.26, Figure 5 shows that the equilibrium increment of the GoHAP-1 (10 nm) mass is five times higher than that of GoHAP-6 (40 nm) (5.2% and 1%, respectively). This result is correlated with the more than fourfold difference between the total surface areas of the tested samples (see Table 1). The values of the specific adsorption of GoHAP™, in turn, differ to a slight extent and are 0.25 and 0.21 mg/m^2^, respectively. 

**Figure 6:**
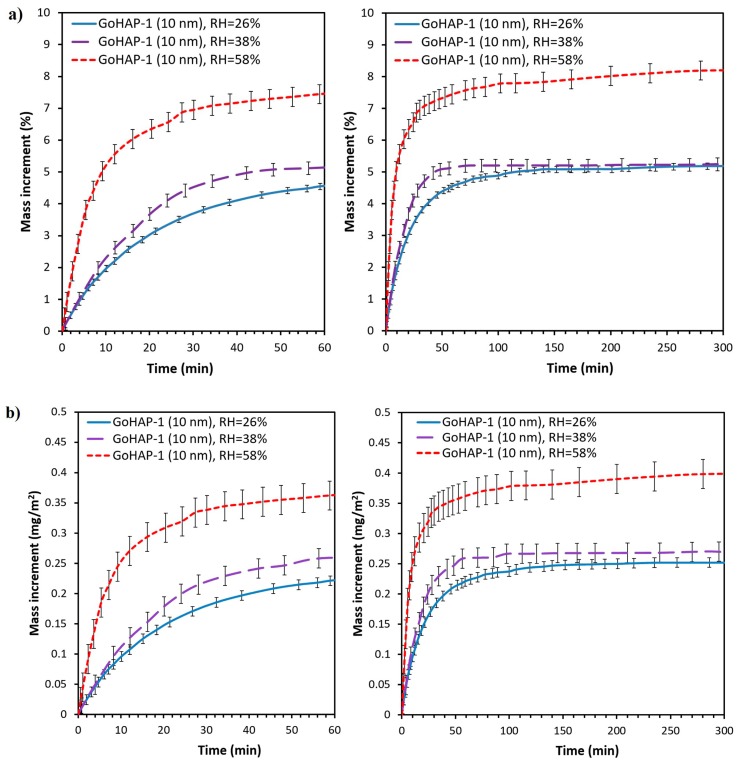
Kinetic curves of water adsorption on GoHAP-1 (10 nm) with varied ambient relative humidity values: (**a**) percentage mass change, (**b**) mass increment per 1 m^2^.

Regarding the impact of the water activity on the kinetics of adsorption, in the case of GoHAP-1 (10 nm), no difference was observed during the initial 10 min of exposure for a relative humidity of 26% and 38%. After that period at the ambient relative humidity of 38%, the mass increase is faster than for a relative humidity of 26% and reaches a state of equilibrium after 70 min. The saturated condition for the relative humidity of 26% is reached after 140 min. Despite differences in the adsorption kinetics, the quantity of water adsorbed by GoHAP-1 (10 nm) at the ambient relative humidity of 26% and 38% is similar and reaches slightly above 5% (Figure 6). At the ambient relative humidity of 58%, in turn, the water adsorption rate for the two initial stages of the process is considerably faster than at the lower relative humidity. Adsorption at the ambient relative humidity of 58% is 8.2%, e.g., 1.5 times higher than for 26% and 38% relative humidity. In other units for the surface, for a relative humidity of 58%, 0.4 mg of H_2_O is bound to 1 m^2^ on the GoHAP™ surface, while for 26% and 38% humidity, the value is about 0.25 mg of H_2_O/m^2^. 

**Figure 7:**
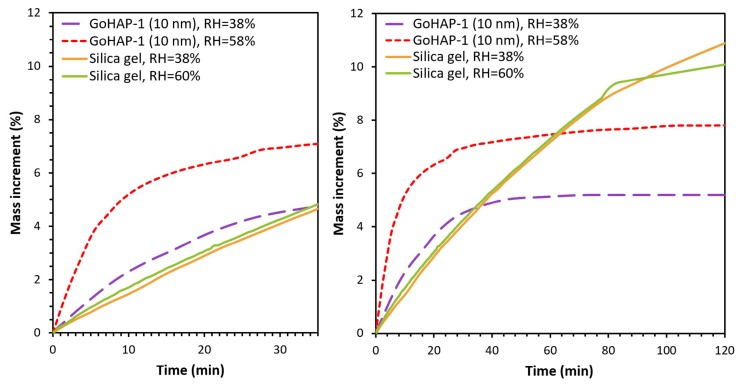
Kinetic curves of water adsorption on GoHAP-1 (10 nm) and Silica gel.

The kinetics of water adsorption of GoHAP™ was compared with a reference sample of silica gel. Samples were exposed for 2 h to the ambient relative humidity of 38 ± 2% and 58 ± 2% (Figure 7).

The silica gel mass did not stabilise within the duration of the experiment, and after two hours of adsorption, it increased by 10–11%. The adsorption rate of the silica gel was the same for both ambient water activity values. The experiment indicated that, at the first stage of the process during the initial 30 min of exposure, GoHAP-1 (10 nm) was characterised by a considerably higher activity compared to silica gel. This result is significant because it shows that GoHAP-1 (10 nm) not only has very good sorption properties, but also can be a more effective and competitive sorbent than silica gel if there is a need to rapidly reduce the relative air humidity. 

**Figure 8:**
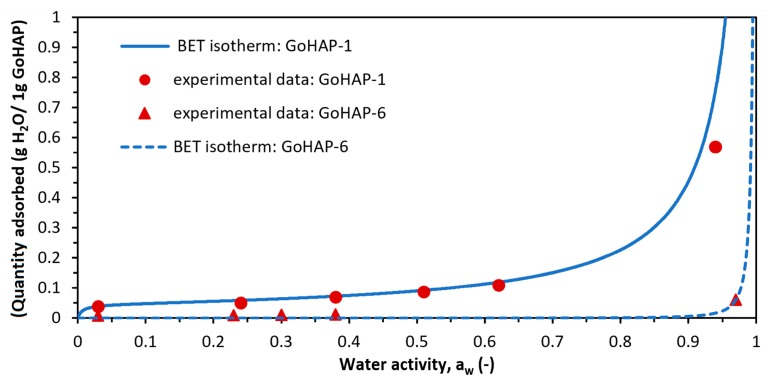
Brunauer–Emmett–Teller (BET) isotherm determined for water adsorption on the surface of GoHAP-1 (10 nm) and GoHAP-6 (40 nm).

Based on the results of kinetic tests, an isotherm of water vapour adsorption on GoHAP-1 (10 nm) and GoHAP-6 (40 nm) was determined. Figure 8 presents the isotherm expressed as a change in the relative increment of GoHAP™ mass as a function of water activity. The experimental curve was compared with the isotherm models described in the relevant literature. It was found that the nature of the adsorption isotherm was consistent with the Brunauer—Emmet—Teller (BET) isotherm model [63,64], described by Equation (3):

(3) $$a=\frac{a_{m}C\frac{p}{p_{0}}}{\left(1-\frac{p}{p_{0}}\right)\left[1+\left(C-1\right)\frac{p}{p_{0}}\right]}$$

where: *a* is the total volume of adsorbed gas under pressure *p* (g H_2_O/g GoHAP); *p*_0_ is the pressure of the adsorbate’s saturated vapour (Pa); *a_m_* is the volume of the monolayer (g H_2_O/g GoHAP); *C* is the adsorption equilibrium constant; and *p*/*p*_0_ = *a_w_* is the water activity.

The parameters for fitting the BET adsorption isotherm to the experimental data were determined (Figure 8, Table 3). However, the equation for this isotherm best fits the low range of pressures.

The GoHAP™ adsorption isotherm is characteristic of type III isotherms according to Brunauer’s classification [64]. This implies that hydrogen bonds are formed between the adsorbent (GoHAP™) and the adsorbate (water) that are stronger than intermolecular interactions such as van der Waals forces. The isotherm type also suggests that a multilayer adsorption process occurs on the GoHAP™ surface. The parameter am (Table 3) corresponds to a capacity of one layer of water molecules adsorbed per 1 gram of GoHAP™. Therefore, knowledge of the am value is needed to calculate the number of water layers under given conditions.

The thickness of the water layer adsorbed on a GoHAP‒1 surface was determined after exposure for 20 h to air with a relative humidity of 3%, 24%, 38%, 51%, 62%, and 94% (Table 4). For GoHAP-6 (40 nm), the thickness of the water layer was revealed for a relative humidity of 3%, 13%, 24%, 30%, 38%, and 97% and an exposure time of 20 h (Table 5). The monolayer water coverage was calculated both by the SSA method of the HA sample [29,31] and by using moisture sorption isotherm data (Figure 8) fitted to the modified BET equation. The mass of one water molecule is 2.99·10^−23^ g, and the cross-sectional area of a water molecule must take into account the packing density between water molecules and the hydroxyapatite surface. The cross-sectional area for the adsorption of a water molecule onto the surface of HAP has previously been reported as 0.115 nm^2^ [29].

The monolayer coating calculated using the modified BET method was lower than that calculated using the surface area approach, similar to calculations [31] for granulated hydroxyapatite. According to the authors of Reference [31], the BET approach will probably underestimate the true coverage of the monolayer, and it can be assumed that a specific approach to the surface can provide a more accurate determination of the monolayer coverage. Therefore, the number of water layers was calculated by dividing the humidity sample content by the monolayer water coverage of 0.2605 mg/m^2^ [29]. The calculation included the packing efficiency of 0.74 [65].

**Figure 9:**
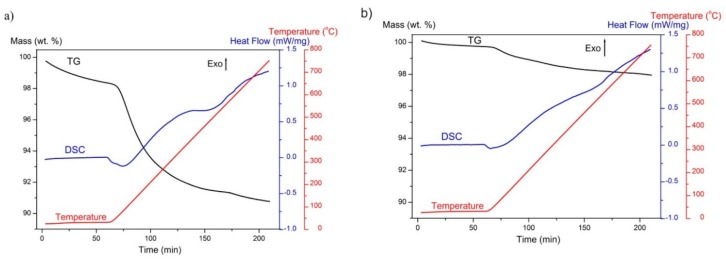
Thermogravimetry (TG) and calorimetry (DSC) curves of (**a**) GoHAP-1 (10 nm) and (**b**) GoHAP-6 (40 nm) soaked up to 800 °C. The temperature change as a function of time is also presented.

**Figure 10:**
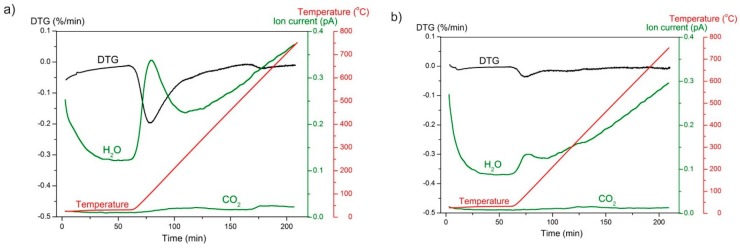
H_2_O and CO_2_ ion currents and derivative thermogravimetric (DTG) curves of (**a**) GoHAP-1 (10 nm) and (**b**) GoHAP-6 (40 nm) soaked up to 800 °C. The temperature change as a function of time is also presented.

The results of a thermogravimetric analysis coupled with differential scanning calorimetry of GoHAP™ are presented in Figure 9. The thermogravimetric (TG) curve reflects the changes in the mass of the sample as a function of temperature. The calorimetry curve (DSC) reflects the heat flow corresponding to the processes occurring in the sample. Mass spectrometry results, presenting ion currents of H_2_O (m/z = 18) and CO_2_ (m/z = 44) in gases released from samples during the TG experiment, are shown in Figure 8. In addition, derivatives of the thermogravimetric curves (DTG) are drawn in Figure 10 to allow a comparison between the analysed ion current changes and the mass changes in the samples.

A sample mass decrease was observed throughout the entire TG experiments. During the isothermal stage at room temperature, the nanoparticle surface was cleaned just as a result of helium flushing. A significant acceleration in the mass change occurs after the beginning of the heating stage. The highest speed of mass loss (seen as a peak on the DTG curve), correlating with an increase in water desorption (reflected as a peak on the H_2_O line), was recorded after 10 min of heating. Intense water desorption occurred up to approximately 150 °C. This is a 150 °C lower temperature than the desorption temperature of microcrystalline hydroxyapatite proposed by Holmes et al. [30]. According to Reference [32], structural water is removed from GoHAP™ in the temperature range 200 °C to 600 °C. In the present work, this phenomenon overlaps with the removal of CO_2_ molecules from hydroxyapatite, resulting in a slight increase of the ionic current value (Figure 10). The sum of both processes was reflected as a small peak on the DTG curve of GoHAP-1 (10 nm) and is more intense on the DSC curves for GoHAP-1 (10 nm) and GoHAP-6 (40 nm) (Figure 9 and Figure 10).

Thermal analysis coupled with mass spectroscopy of both hydroxyapatite sizes showed that the recorded mass loss results mostly from dehydration. However, it also clearly revealed significant differences between the rate and effectiveness of water desorption depending on nanoparticle size. While flushing the GoHAP-1 (10 nm) with helium at room temperature for 1 h, the mass loss was 1.64 wt %. The weight loss resulting from heating to 800 °C was 7.59 wt %. In total, the GoHAP-1 (10 nm) mass loss was 9.23 wt %. For GoHAP-6 (40 nm), the helium flush stage resulted in a mass loss of 0.26%, and heating the sample to 800 °C resulted in 1.78% of the mass being lost. In total, the mass of GoHAP-6 (40 nm) decreased by 2.04%. Mass changes during the TG process are similar to those obtained in the desorption station as a result of heating for 2.5 h at 150 °C and flushing with helium (Table 6). This demonstrates that an appropriate selection of the sample desorption conditions leads to nearly total degassing of GoHAP™ without high temperature treatment.

## 4. Discussion

**Figure 11:**
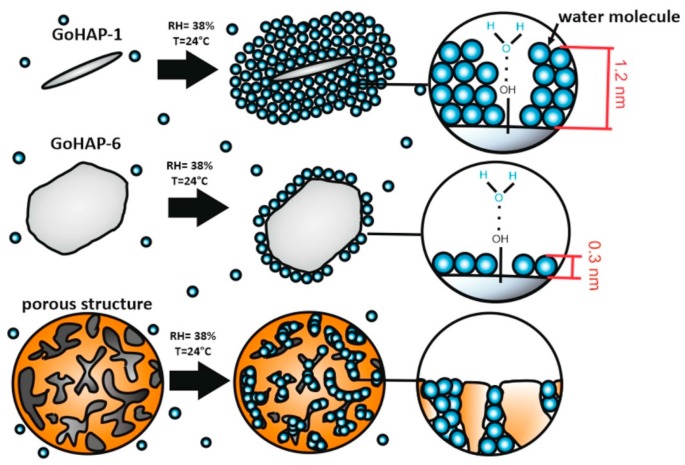
Nanoparticle size and shape effect on water adsorption.

The mechanism of water binding by 10 nm nano-hydroxyapatite, 40 nm nano-hydroxyapatite, and microcrystalline hydroxyapatite [30] is the same regardless of the particle size. However, the size of hydroxyapatite affect the amount of adsorbed water. With a relative humidity between 3–97%, many more layers/molecules of water are adsorbed on the surface of 10 nm GoHAP™ than 40 nm GoHAP™. The greater increment in the 10 nm GoHAP™ mass is probably caused by two factors: (1) the higher availability of the surface that water molecules can directly attach to, and (2) the larger number of monolayers. Nanoparticles have a large ratio of surface area to mass. Nanoparticles with dimensions of several nm are composed largely of surface atoms and contribution of surface energy to their total free energy is high [66]. An increase of diameter reduces the contribution of surface energy to the total energy and this is the driving force for adsorption of several monolayers of water. This explains why the 10 nm particles absorb more monolayers of water than the 40 nm ones (Figure 11). Therefore, the shape and size of GoHAP™ determine the amount of water adsorbed on the surface. The deposition of the first water layer on the surface of GoHAP™ begins at places where free OH groups are present on the surface of the crystals. The molecules of the first layer of water are strongly bound to the surfaces of nano-, micro- [28,29], and natural [28,29,30] hydroxyapatite by means of hydrogen bonds formed between hydroxy groups of hydroxyapatite and water molecules. Subsequent layers are attached by cohesion forces due to the formation of weak hydrogen bonds between water molecules. With subsequent layers of the water molecule, the Sauther mean diameter of the individual particles increases and the specific surface area decreases. The Sauter mean diameter is increased until a critical value is attained for further attachment of the water molecule layers under given ambient conditions. It can also be assumed that the amount of adsorbed water is limited because the water layers of the nano-hydroxyapatite start to contact at larger Sauther diameter, further decreasing the specific surface area available for water adsorption.

The state of dispersion of hydroxyapatite can also affect the adsorption process. The studies presented earlier [31] state that the amount of water adsorbed by HAP granulate with a specific surface area of 60 m^2^/g is 5.26% at 25 °C and RH = 30%. This result is similar to the amount of water adsorbed by loosely scattered GoHAP™ with a specific surface area of 206 m^2^/g under similar ambient conditions. Such results may indicate a high porosity of the granules. Considering this, it can be assumed that water molecules are not only adsorbed on the surface of granular hydroxyapatite, but also that most of them are trapped in pores of granules formed between individual HAP particles. However, the rates of adsorption on the surface of loosely scattered HAP. 

A comparison shows that water adsorption on the surface of 10 nm GoHAP™ is faster than that for silica gel granules. The rate of water adsorption by 10 nm GoHAP™ nanoparticles is the highest during the first 30–50 min of contact with air at 23 °C and at relative humidity levels of 38% and 58%, respectively (Figure 7). GoHAP™ nanoparticles connect with water faster from the environment and reach equilibrium faster. The higher the air humidity, the higher the GoHAP™ adsorption rate. Meanwhile, silica gel granules bind water at a constant rate regardless of the humidity level, and their saturation occurs later. Silica gel adsorbs water more slowly, despite higher surface development compared to 10 nm GoHAP™ particles. Presumably water molecules must reach the inside of the pores of the silica gel, which slows the water adsorption process in relation to the adsorption on the surface of the loosely spilled particles. Despite the slower course of the adsorption process, the final amount of adsorbed water is higher for silica gel granules.

These results are important for applications. The relationship between the size of nano-hydroxyapatite and the amount of adsorbed water allows the water content in the material to be regulated, by adjusting the ambient humidity, exposure time and nanoparticles size. There are many applications in which a few percent difference in water content is crucial for material properties. For example, ibuprofen tablets have a maximum tensile strength at a water content of about 2.5%, but this is significantly reduced at a water content of 10 wt % [44]. A similar change in material properties under the influence of moisture content was observed for anhydrous dextrose—an increase in water content from 0.34 to 8.9 wt % resulted in an increase in the tensile strength of the tablet, whereas a water content exceeding 9.2 wt % resulted in a significant reduction in tensile strength [67]. For this reason, the choice of nano-hydroxyapatite for specific applications should consider the particle size, as this significantly affects the amount of adsorbed water in the mass of material. Another factor influencing the water content on the surface of nano-hydroxyapatite is the relative humidity of the air. Therefore, the user of nano-HAP should pay special attention to handling and storage conditions.

## 5. Conclusions

Water adsorption and desorption by hydroxyapatite were tested as a function of nanoparticle size and the ambient relative air humidity. Two types of hydroxyapatite nanopowders obtained by microwave hydrothermal synthesis were tested. The mean nanoparticle sizes were 10 and 40 nm, and the specific surface areas were 206 m^2^/g and 49 m^2^/g, respectively.

Nanoparticle size has a strong effect on the kinetics and efficiency of water adsorption. For the same value of water activity in air, the quantity of water adsorbed on the surface of 10 nm hydroxyapatite was five times greater than that adsorbed on the 40 nm particles. It was found that the multilayer physical adsorption mechanism was active. The number of H_2_O molecules layers adsorbed depended on air humidity and particles size. For 10 nm particles it ranged from 1.5 to 23.6 monolayers for water activity in the range 0.03–0.94. For particles of 40 nm size, it ranged from 0.6 to 4.8 monolayers in the same humidity range. The 10 nm needle-shaped particles have water adsorption properties comparable with silica gel. During the initial 30 min of sample contact with humid air, the water adsorption of 10 nm hydroxyapatite was even more effective than it is for silica gel. Saturation of water adsorption was observed after approximately 100 min of contact between the nanoparticles and air, regardless of the humidity level.

These findings are important for optimising the processing of hydroxyapatite and other nanoparticles in environment of varying humidity.

## Figures and Tables

**Table 1:** GoHAP™ characterisation. Specific surface area (SSA); skeleton density (DEN); mean particle size based on BET (SMD_BET_); TEM histogram of log-normal curve (MPS_TEM_); mean crystallite size from Nanopowder XRD Processor Demo (MCS_XRD_) [41,44]; Scherrer equation (MCS_Sch_).

Sample Name	SSA (BET)	DEN	SMD_BET_	MPS_TEM_	MCS_XRD_	MCS_Sch_	
	(m^2^/g)	(g/cm^3^)	(nm)	(nm)	(nm)	Length (nm)	Width (nm)
GoHAP-1 (10 nm)	206 ± 1	2.87 ± 0.01	10	-	10 ± 6	19	5
GoHAP-6 (40 nm)	49 ± 1	3.09 ± 0.01	40	39 ± 1	62 ± 43	54	34

**Table 2:** Impact of ambient relative humidity on the change in mass of GoHAP-1 (10 nm) samples.

Relative Humidity (%)	Adsorption (%)	Specific Adsorption (mg/m^2^)
26	5.2	0.25
38	5.4	0.26
58	8.2	0.40

**Table 3:** Parameters of BET isotherm of water adsorption on the surface of GoHAP-1 (10 nm) and GoHAP-6 (40 nm). The volume of the monolayer (a_m_ (g H_2_O/ g GoHAP)); the adsorption equilibrium constant (C); standard deviation (S); Fisher Test (F-test).

Test Sample	Parameter		Statistics	
	a_m_	c	S	F-test
GoHAP-1 (10 nm)	0.045	161	0.013	230
GoHAP-6 (40 nm)	0.0078	0.00419	0.011	3.72

**Table 4:** Estimated water layer thickness and number of monolayers of H_2_O molecules (n_H2O_) and mean water layer thickness (h_H2O)_ adsorbed on the surface of GoHAP-1 (10 nm), depending on experimental conditions and an exposure time of 20 h.

Relative Humidity	$h_{H_{2}O\;}\;(\mathrm{nm})$		$n_{H_{2}O}$	
	from SSA Method	from BET Isotherm	from SSA Method	from BET Isotherm
3%	0.5	0.3	1.5	0.9
24%	1.1	0.3	3.8	1.6
38%	1.2	0.6	4.1	2
62%	1.3	0.9	4.4	3
94%	7.1	3.6	23.6	12

**Table 5:** Water layer thickness and number of monolayers of H_2_O molecules adsorbed on the surface of GoHAP-6 (40 nm), depending on the experimental conditions and an exposure time of 20 h.

Relative Humidity	$h_{H_{2}O}\;(\mathrm{nm})$		$n_{H_{2}O}$	
	from SSA Method	from BET Isotherm	from SSA Method	from BET Isotherm
3%	0.2	0.3	0.6	1.0
24%	0.2	0.4	0.7	1.2
30%	0.3	0.5	0.9	1.5
38%	0.3	0.5	1.0	1.6
97%	1.4	2.3	4.8	7.8

**Table 6:** Mass changes in GoHAP™ during thermogravimetric analysis (TG) and in the desorption station (DS).

Sample Name	Mass Change (%)	
	Heating to 800 °C (TG)	Heating to 150 °C (DS)
GoHAP-1 (10 nm)	−9.2	−6.3
GoHAP-6 (40 nm)	−2.0	−1.8

## Supplementary Materials

The following are available online at https://www.mdpi.com/2079-4991/9/7/1005/s1, Figure S1: Nitrogen adsorption isotherms of GoHAP™. STP means standard temperature and pressure; Table S1: Characteristics of GoHAP™ samples; Table S2: Characteristics of GoHAP™ samples.
