# Supplementary material for: Nanoparticle Size Effect on Water Vapour Adsorption by Hydroxyapatite
Source: Nanomaterials (Basel). 2019 Jul 12;9(7):1005. doi: 10.3390/nano9071005 (PMC6669690; doi:10.3390/nano9071005)
Supplement: Supplementary file 1 [file nanomaterials-09-01005-s001.pdf]

Supplementary Materials

Nanoparticle Size Effect on Water Vapour Adsorption by Hydroxyapatite

Urszula Szałaj [1,2,*], Anna Świderska-Środa [1], Agnieszka Chodara [1,2], Stanisław Gierlotka [1] and Witold Łojkowski [1]

1 Institute of High Pressure Physics, Polish Academy of Sciences, Sokołowska 29/37, 01-142 Warsaw, Poland
2 Faculty of Materials Engineering, Warsaw University of Technology, Wołoska 41, 02-507 Warsaw, Poland
* Correspondence: u.szalaj@labnano.pl; Tel.: +48-22-876-04-31

The average pore width was calculated by the Barrett-Joyner-Halenda (BJH) method using adsorption isotherms at the P/P0 range of 0.001–0.99. The Adsorption Isotherm was determined using the helium pycnometer at the temperature of 24 ± 1 °C (ISO 12154:2014, AccuPyc II 1340 FoamPyc V1.06, Micromeritics, USA). The total pore volume of samples was estimated from the amount of adsorbed nitrogen at P/P_0 = 0.9896. The obtained data of the adsorption isotherms were analysed by us using the MicroActive software V4.03 (Interactive Data Analysis Software, Micromeritics).

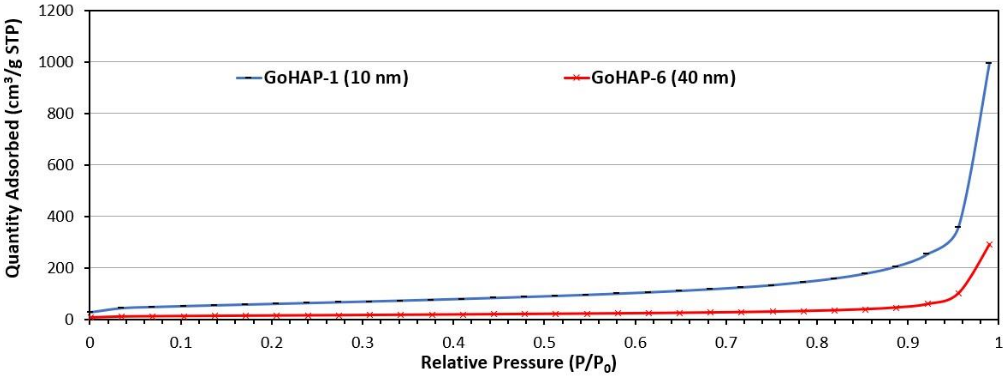

Figure S1. Nitrogen adsorption isotherms of GoHAP™. STP means standard temperature and pressure.

Table S1. Characteristics of GoHAP™ samples.

Sample	Specific Surface Area by Gas Adsorption, $a_s \pm \sigma$ (m^2/g)	BJH Adsorption Average Pore Width (Å)	Total Pore Volume at P/P_0 = 0.9896 (cm^3/g)	Skeleton Density by Gas Pycnometry, $\varrho_s \pm \sigma$ (g/cm^3)
GoHAP-1 (10 nm)	209 ± 1	292.2	1.538	2.87 ± 0.01
GoHAP-6 (40 nm)	49.7	319.9	0.451	3.09 ± 0.01

Table S2 shows the results of the Specific Surface Area and Skeleton Density measurement before and after the Adsorption-Desorption-Adsorption-Desorption cycle (A-D-A-D cycle).

Table S2. Characteristics of GoHAP™ samples.

Sample Name	Specific Surface Area by Gas Adsorption SSA (m^2/g)	Skeleton Density by Gas Pycnometry, DEN ± σ (g/cm^3)
GoHAP-1 (10 nm)	206 ± 1	2.87 ± 0.02
GoHAP-1 (10 nm) after A-D-A-D cycle	209 ± 1	2.92 ± 0.02
GoHAP-6 (40 nm)	49 ± 1	3.09 ± 0.01
GoHAP-6 (40nm) after A-D-A-D cycle	49 ± 1	3.09 ± 0.01
